# Physical Therapists and Physical Therapist Assistants’ Knowledge and Use of the STEADI for Falls Risk Screening of Older Adults in Physical Therapy Practice in the United States

**DOI:** 10.3390/ijerph19031354

**Published:** 2022-01-26

**Authors:** Jennifer L. Vincenzo, Lori A. Schrodt, Colleen Hergott, Subashan Perera, Jennifer Tripken, Tiffany E. Shubert, Jennifer S. Brach

**Affiliations:** 1 Department of Physical Therapy, University of Arkansas for Medical Sciences, Fayetteville, AR 72701, USA; 2 Department of Physical Therapy, Western Carolina University, Cullowhee, NC 28723, USA; lschrodt@email.wcu.edu; 3 Department of Physical Therapy, Augusta University, Augusta, GA 30912, USA; chergott@augusta.edu; 4 Department of Medicine, University of Pittsburgh, Pittsburgh, PA 15260, USA; ksp9@pitt.edu; 5 Center for Healthy Aging, National Council on Aging, Washington, DC 22202, USA; jennifer.tripken@ncoa.org; 6 UNC Center for Health Promotion and Disease Prevention, University of North Carolina, Chapel Hill, NC 27514, USA; tiffany.shubert@gmail.com; 7 Department of Physical Therapy, University of Pittsburgh, Pittsburgh, PA 15260, USA; jbrach@pitt.edu

**Keywords:** evidence-based practice, accidental injury, injury prevention, rehabilitation, health services, preventive healthcare

## Abstract

Fall-risk screening and prevention is within the scope of physical-therapy practice. Prior research indicates United States-based physical therapists (PTs) and physical-therapist assistants (PTAs) use the Centers for Disease Control and Prevention’s STEADI (Stopping Elderly Accidents, Deaths, and Injuries) toolkit for community-based fall-risk screenings of older adults. However, clinically based fall-risk screenings and knowledge and use of the STEADI by PTs and PTAs is unknown. We conducted a cross-sectional survey distributed to a convenience sample of PTs and PTAs in the United States through email blasts and social media. PTs and PTAs (N = 425) who responded to the survey and worked in clinical settings with older adults were included. Eighty-nine percent of respondents reported conducting clinical fall-risk screening. Approximately 51% were ‘familiar’ to ‘very familiar’ with the STEADI, and 21.7% of the overall sample were not familiar at all. Only 26.1% utilize the STEADI for clinical fall-risk screening. Of the respondents who were ‘very familiar’ with the STEADI (*n* = 132, 31.1%), 84.1% (*n* = 111) reported using the STEADI in clinical practice. Seventy-six percent of respondents who use the STEADI implemented it by choice even though the majority (52.1%, *n* = 63) did not have it embedded in their documentation/workflow. Some PTs/PTAs can and do manage falls using the STEADI, but there is a gap in knowledge and use of the STEADI for falls management among PTs and PTAs in the United States. Further research is needed to identify the tools PTs use for multifactorial-fall screening and management and the impact of PTs’ use of the STEADI on patient outcomes.

## 1. Introduction

**Figure 1:**
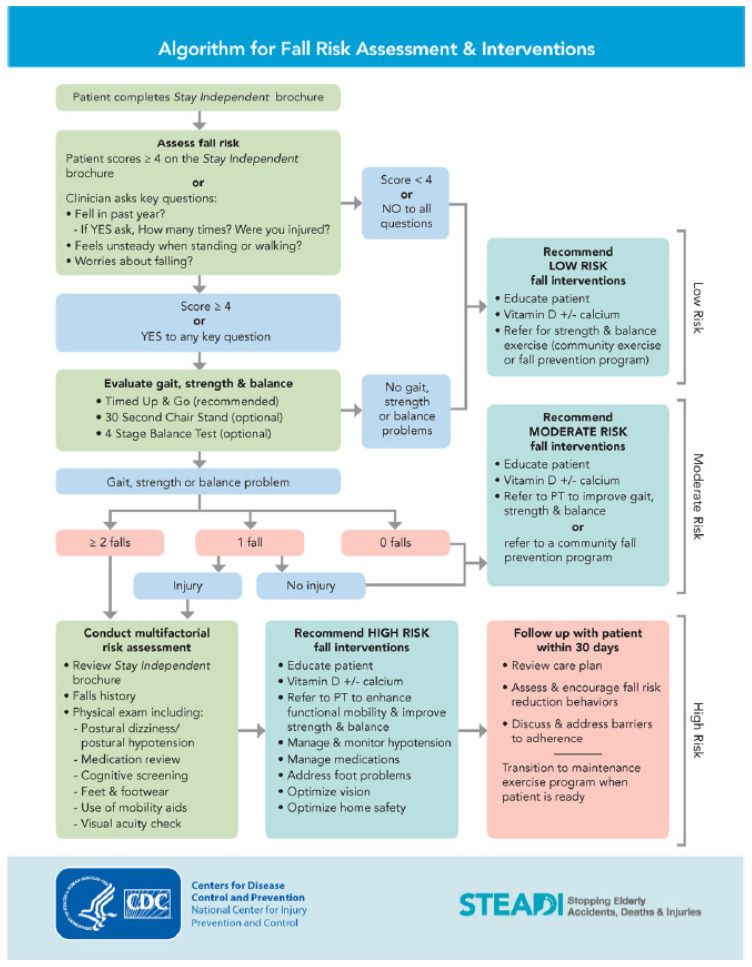
The STEADI algorithm from 2019 [5,6].

Falls are the leading cause of fatal and nonfatal injuries among aging adults, costing upwards of USD 50 billion in yearly healthcare costs [1]. Many falls are preventable with screening to identify older adults at risk, followed by multifactorial assessments and interventions to ameliorate risks [1]. Physical therapists (PTs) and physical-therapist assistants (PTAs) are critical members of the healthcare team involved in fall prevention, most notably assessing balance and gait and providing exercise interventions for older adults at risk [2]. However, Gell and colleagues found that only 50% of older adults undergoing rehabilitation who were at risk of falls reportedly had falls addressed during rehabilitation [3]. This data indicates a gap in either knowledge or practice of fall prevention among PTs/PTAs. The Clinical Guidance Statement from the Academy of Geriatric Physical Therapy of the American Physical Therapy Association (APTA) recommends that PTs should screen all older adults annually for fall-risk and assess and intervene for those identified at risk [4]. The Centers for Disease Control and Prevention developed the STEADI (Stopping Elderly Accidents, Deaths, and Injuries) toolkit to promote evidence-based screening, assessment, and interventions to reduce falls among older adults [5]. The STEADI was developed for primary care providers; however, the toolkit can be used by any healthcare professional in any setting. The toolkit has numerous online training modules, documents, and other information for healthcare providers and older adults to prevent falls [5]. The 2019 STEADI (referred to in this study) follows an algorithm of screening and multifactorial assessment classifying an older adult as having low, moderate, or high risk of falls. The algorithm then lists interventions and referrals to address risk factors corresponding to each level of risk (Figure 1) [6]. There are a multitude of modifiable factors that increase an older adult’s risk of falling included in screening and/or multifactorial assessment within the STEADI. These include history of falls, fear of falling, decreased strength or balance, unsteady gait, low blood pressure, visual or memory impairments, foot problems, issues with safety in the home, and side effects of some medications [1,2,4,5]. Fall-risk screening and prevention are within the scope of physical-therapy practice, and several studies indicate that PTs and PTAs in the United States (US) use the STEADI in community-based fall-risk screenings for older adults [6,7,8]. To our knowledge, no studies have investigated PTs and PTAs in the US self-reported-clinical fall-risk-screening practices and knowledge and use of the STEADI in clinical settings. These data may provide valuable information regarding gaps in fall-risk management identified in the literature. Therefore, we aimed to identify US PTs’ and PTAs’ engagement in clinical fall-risk screenings of older adults and knowledge and use of the STEADI.

## 2. Materials and Methods

The study was a cross-sectional survey of a convenience sample of PTs and PTAs in the US conducted in 2019. The study was deemed exempt after review by the Institutional Review Board; therefore, informed consent was not obtained. However, information and details regarding the purpose, voluntary nature of the survey, and anonymity of responses were presented to potential participants before initiating the survey.

### 2.1. Survey

A task force of fall-prevention experts (APTA Geriatrics and the National Council on Aging [NCOA]) developed and piloted a 36-item, 20 min, web-based, cross-sectional survey to identify fall-risk-screening practices and the STEADI knowledge and use among PTs and PTAs. The STEADI algorithm from 2019 is presented in Figure 1. Reliability was not determined. The cross-sectional survey was administered through REDCap (Research Electronic Data Capture [9] hosted by the University of Arkansas for Medical Sciences. The survey was disseminated, and data were collected from a convenience sample of PTs and PTAs from September to November 2019 via email, e-blasts, and social media. These electronic recruitment methods were sent to sections and academies in the APTA, posted on physical-therapy specific social media sites, and shared by physical therapists and assistants on social media and by email. The survey was not password-protected to enable others to share the link with colleagues. Responses were anonymous. The inclusion criterion was PTs or PTAs in any practice setting involved in the clinical care of older adults in the US. The exclusion criterion was anyone not actively involved in clinical care of older adults. Incomplete surveys were excluded from the analyses.

### 2.2. Statistical Analysis

Descriptive statistics were used to summarize the demographic characteristics of the respondents. Some categorical variables were combined to provide more meaningful classifications or due to small frequencies. We used the independent-samples t-test for continuous data and the chi-square and Fisher’s exact tests for categorical data to compare characteristics between respondents that do and do not conduct fall-risk screenings. Frequency counts and percentages were used to summarize survey responses related to fall-risk screening and knowledge/use of the STEADI. SAS^®^ version 9.4 (SAS Institute, Inc., Cary, NC, USA) was used for all statistical analyses.

## 3. Results

Four hundred fifty-nine PTs and PTAs from 49/50 states in the US across various settings participated. Respondents who did not complete the survey or meet the inclusion criterion were removed from the data (*n* = 34), leaving 425 respondents for analysis. Approximately 90% of respondents (*n* = 377) were PTs and approximately 11% (*n* = 48) were PTAs. Seventy-five percent of respondents were members of the APTA. Almost half had over 20 years of experience (48.5%), worked in an outpatient/wellness setting (47.5%), and had a caseload of 80–100% of older adults (62.8%). Approximately 45% of respondents held a board certification, indicating advanced practice in physical therapy, with the majority in geriatrics (32.2%). Demographics of respondents by engagement in clinical fall-risk screenings are presented in Table 1. Eighty-nine percent of respondents indicated they conduct fall-risk screenings for adults over the age of 65 years. An approximately equal frequency of respondents (~32%) indicated they screen between 26–100 or 101–500 older adults annually for fall risk.

Table 2 depicts the knowledge and implementation of the STEADI in clinical practice. Approximately 51% of survey respondents were ‘familiar’ to ‘very familiar’ with the STEADI, while 21.7% were ‘not familiar at all’. Of the respondents who were ‘very familiar’ with the STEADI (*n* = 132, 31.1%), 84.1% (*n* = 111) reported using it in clinical practice, which is only 26.1% of the entire sample (*n* = 425). PTs and PTAs reported using all components of the STEADI but utilizing the education and intervention components within the algorithm the most (86.1%) and the Stay Independent Brochure and Questionnaire the least (46.7%). Seventy-six percent of therapists who use the STEADI implemented it by choice. Of the PTs/PTAs who use the STEADI, the majority (52.1%, *n* = 63) do not have it embedded in their documentation/workflow.

## 4. Discussion

Fall-risk screening is the first component of multifactorial-fall prevention. Our research is the first to identify US PTs’ and PTAs’ reported engagement in fall-risk screening and knowledge and use of the STEADI in clinical practice. We found that 89% of therapists who responded to this survey reported conducting fall-risk screenings on older adults. An approximately equal frequency of respondents (~32%) indicated they screen between 26–100 or 101–500 older adults annually. However, considering that ~85 % of the respondents’ caseloads consisted of adults over the age of 65 years, it appears that PTs and PTAs may not be screening *all* older adults for falls despite the APTA-Geriatrics clinical-guidance statement maintaining that PTs and PTAs should conduct fall-risk screenings on *all* older adults annually [4]. These findings are supported by Gell and colleagues’ study that found that only 50% of older adults undergoing rehabilitation who were at risk of falls reportedly had falls addressed during their rehabilitation [3]. Future studies will be beneficial to identify the barriers and facilitators to implementing fall management in routine rehabilitation among older adults.

Although 89% of PTs/PTAs reported conducting fall-risk screenings with older adults, only half were ‘familiar’ to ‘very familiar’ with the STEADI. Considering the STEADI is one of the most disseminated toolkits in the US for fall prevention [5], the lack of awareness warrants further research regarding which tools PTs/PTAs use to screen, assess, and manage older adults for fall risk. Among 370 Australian PTs who work with patients with hip and knee osteoarthritis, discrepancies were noted in the therapists’ knowledge of fall prevention and use of appropriate tools to screen for fall risk. Although 84% of the PTs reported having fall-related education, 61% reportedly did not use fall-risk screening tools [10]. The PTs who reported using screening tools used a wide variety of tools. These findings may be because PTs are experts in physical-function and outcome measures and have many evidence-based tools available to measure fall risk in older adults. A recent systematic review by Lusardi et al. found that 56 self-report and functional–outcome measures for community-dwelling older adults were predictive of falls [11]. Therefore, a PT with current knowledge of evidence-based practices would have numerous validated measures to choose from for fall-risk screening and assessment.

Despite having many options, the majority of PTs and PTAs in this study who were very familiar with the STEADI used the toolkit for fall-risk screening and management in clinical practice by choice rather than employer mandate, even though over half did not have the tool embedded in their documentation/workflow. Previous research indicates lack of employer mandate [12] or lack of integrated workflows in electronic medical records [13] are barriers to implementation of evidence-based practice. Despite these potential barriers and the number of validated clinical fall-risk screenings and assessments available, PTs’ use of the STEADI suggests that those very familiar with the tool find it appropriate and valuable for fall-risk management of older adults. Further research is necessary to identify which screening and assessment measures US PTs/PTAs use for fall risk and which factors affect use of the STEADI.

PTs and PTAs who reported using the STEADI toolkit utilized the education/interventions within the algorithm the most, followed by the three key questions. Those who used the toolkit reported using the Stay Independent Brochure and Questionnaire the least. The three key questions may be utilized more than the questionnaire because asking an older adult three questions (i.e., feels unsteady, worries about falling, has fallen in the past year) is a quicker way for clinicians to screen for fall risk compared to the 12-item, self-report Stay Independent Brochure and Questionnaire [5]. Although our study focuses on screening, our findings suggest that those who reported using the STEADI for screening are likely providing interventions to manage fall risk because they report using the education and interventions within the algorithm the most out of all items in the toolkit.

This study has several strengths. To our knowledge, this is the first study to report US PTs’ and PTAs’ engagement in clinical fall-risk screening and knowledge and use of the STEADI. The survey dissemination strategy resulted in the representation of therapists from 49/50 states in the US from various physical-therapy-practice settings. However, this study does have limitations. Although our dissemination strategy allowed for a broad reach, we could not determine how many PTs or PTAs received the survey or the response rate. Fewer PTAs completed the survey than PTs, which may decrease the generalizability of the results for PTAs. In addition, more than ¾ of the survey respondents were APTA members, compared to less than ¼ of all licensed PTs/PTAs nationally, and almost half of our respondents were board-certified, compared to 12% of all licensed PTs. Considering that APTA members have more access to evidence-based practice documents and that board certification indicates advanced practice, it is likely the number of PTs and PTAs who screen and are aware of and use the STEADI is lower nationally than what is represented in our results.

Another possible limitation of our survey is that we did not specifically define screening. Screening is a common term in physical therapy and is embedded in the history and examination process [14]. Screening knowledge and proficiency are required elements of entry-level curricula, and familiarity with the term screening was assumed among survey participants [15]. Evidence supports varied valid approaches to fall-risk screenings, such as history questions, questionnaires, and performance-based measures of balance and walking based on various functional abilities and care settings [4,11]. Thus, although screening older adults for fall risk is recommended by evidence-based guidelines, the specific tool for screening is most often based on clinical judgment with consideration for individual patient abilities. There are opportunities for future efforts to identify which screening and assessment tools PTs and PTAs use, barriers PTs have conducting multifactorial-fall prevention in different settings, and the need for implementation studies similar to those conducted using the STEADI in primary care. These lines of research may support the achievement of older adults receiving fall-risk screening and prevention.

## 5. Conclusions

The results of our study are the first to identify a majority of PTs and PTAs in the US who responded to this survey reported conducting clinical fall-risk screening of older adults, and approximately half of those who are screening for falls have some knowledge of the STEADI. However, only one-fourth of PTs/PTAs utilize the STEADI for clinical fall-risk screening, with the majority of individuals who use the tool being those who were ‘very familiar’ with it. Three-fourths of respondents who use the STEADI implemented it by choice even though the majority did not have it embedded in their documentation/workflow. Previous research supports PTs and PTAs as critical members of the multidisciplinary team for falls prevention to assess and improve mobility, strength, and balance. However, our study indicates PTs can and do conduct multifactorial-fall management. The results of our study indicate there is a gap in knowledge and use of the STEADI for fall management among PTs and PTAs in the US. Further research is needed to identify the tools PTs use for multifactorial-fall screening and fall management and to promote PTs and PTAs playing a more significant role in multifactorial-fall prevention as part of routine physical-therapy care of older adults.

## Figures and Tables

**Table 1:** Demographics of physical therapists and physical-therapist assistants by clinical-fall-risk-screening status (N = 425).

	Conduct Fall-Risk Screening Mean ± SD or *n* (%) (*n* = 379)	Does Not Conduct Fall-Risk Screening Mean ± SD or *n* (%) (*n* = 46)	*p*-Value
Age	46.9 ± 11.7	48.4 ± 11.9	0.4032
Gender			0.1461
Male	65 (17.2)	8 (17.4)	
Female	311 (82.1)	36 (78.3)	
Prefer not to report	3 (0.8)	2 (4.4)	
Occupation			0.0605
Physical therapist	340 (89.7)	37 (80.4)	
Physical therapist assistant	39 (10.3)	9 (19.6)	
Degree			0.59
Associate	24 (6.3)	6 (13.0)	
BS	67 (17.7)	9 (19.6)	
MS	67 (17.7)	8 (17.4)	
DPT	181 (47.8)	18 (39.1)	
EdD/PhD	30 (7.9)	4 (8.7)	
other	10 (2.6)	1 (2.2)	
Years in practice			0.277
≤5	38 (10.0)	8 (17.4)	
6–10	71 (18.7)	5 (10.9)	
11–20	88 (23.2)	9 (19.6)	
>20	182 (48.0)	24 (52.2)	
APTA member			0.5234
Yes	286 (75.5)	33 (71.7)	
No	87 (23.0)	13 (28.3)	
N/A	6 (1.6)	0 (0.0)	
APTA academy/section member			
Geriatrics	229 (60.4)	21 (45.7)	0.0546
Neurologic	81 (21.4)	6 (13.0)	0.1862
Orthopedic	40 (10.6)	7 (15.2)	0.3409
Home health	44 (11.6)	2 (4.4)	0.2054
Health policy and administration	11 (2.9)	2 (4.4)	0.6407
Acute care	29 (7.7)	2 (4.4)	0.5581
Aquatic	4 (1.1)	1 (2.2)	0.4377
Cardiovascular/pulmonary	15 (4.0)	0 (0.0)	0.3882
Electrophysiology and wound	11 (2.9)	1 (2.2)	1
Education	40 (10.6)	5 (10.9)	1
Federal	4 (1.1)	2 (4.4)	0.1297
Hand and upper extremity	1 (0.3)	0 (0.0)	1
Oncologic	10 (2.6)	1 (2.2)	1
Pediatric	3 (0.8)	2 (4.4)	0.0928
Private practice	7 (1.9)	2 (4.4)	0.2532
Research	17 (4.5)	1 (2.2)	0.7069
Sports	2 (0.5)	4 (8.7)	0.0015
Women’s health	10 (2.6)	0 (0.0)	0.6097
Board-certified clinical specialist	176 (46.4)	15 (32.6)	0.075
Cardiovascular and pulmonary	0 (0.0)	1 (2.2)	0.1082
Geriatrics	129 (34.0)	8 (17.4)	0.0225
Neurology	32 (8.4)	1 (2.2)	0.2365
Oncology	1 (0.3)	0 (0.0)	1
Orthopedics	11 (2.9)	5 (10.9)	0.0209
Sports	3 (0.8)	0 (0.0)	1
Women’s health	0 (0.0)	0 (0.0)	-----
Clinical electrophysiology	0 (0.0)	0 (0.0)	-----
Pediatrics	1 (0.3)	0 (0.0)	1
Practice setting			
Outpatient/wellness	178 (47.0)	24 (52.2)	0.5042
Acute care	53 (14.0)	8 (17.4)	0.5337
Assisted living	60 (15.8)	6 (13.0)	0.622
Inpatient rehab	28 (7.4)	1 (2.2)	0.3465
Skilled nursing facility	132 (34.8)	15 (32.6)	0.765
Home health	86 (22.7)	3 (6.5)	0.0113
Academic program	37 (9.8)	5 (10.9)	0.7938
Other	32 (8.4)	2 (4.4)	0.5621
Employment status			0.4846
Full-time	308 (81.3)	40 (87.0)	
Part-time/per diem/other	70 (18.5)	6 (13.0)	
No response	1 (0.3)	0 (0.0)	
Percent of time in patient care			0.1567
0–25%	96 (25.3)	17 (37.0)	
30–50%	36 (9.5)	4 (8.7)	
55–75%	67 (17.7)	3 (6.5)	
80–100%	180 (47.5)	22 (47.8)	
Percent of caseload 65+ years			0.0004
0–25%	17 (4.5)	6 (13.0)	
30–50%	33 (8.7)	6 (13.0)	
55–75%	83 (21.9)	7 (15.2)	
80–100%	244 (64.4)	23 (50.0)	
No response	2 (0.5)	4 (8.7)	

Note. Percentages are rounded to one decimal place and may total greater than 100% due to rounding. American Physical Therapy Association (APTA).

**Table 2:** Knowledge and use of the STEADI to conduct fall-risk screenings in clinical practice (N = 425).

How Familiar Are You with the STEADI as a Tool for Fall-Risk Screening?	*n* (%)
Very familiar	132 (31.1)
Familiar	84 (19.8)
Somewhat familiar	116 (27.3)
Not familiar at all	92 (21.7)
No response	1 (0.2)
**How did you learn about STEADI? (** **Could select more than 1)**	*n* (%)
School	30 (7.1)
Colleague	93 (21.9)
Continuing education	145 (34.1)
Other	79 (18.6)
Not aware of STEADI	15 (3.5)
Other	12 (2.8)
**Do you use the STEADI tool for fall-risk screening?** **(Question only posed to respondents who indicated they were very familiar with the STEADI) (*n* = 132)**	
Yes	111 (84.1)
No	11 (8.3)
Other	10 (7.6)
**What components of the STEADI do you use? (Could select more than 1 answer)** **(Question only posed to respondents who answered ‘Yes’ or ‘Other’ to using the STEADI) (*n* = 122)**	
Screening—Stay Independent Brochure and Questionnaire	57 (46.7)
Screening—3 key questions	89 (72.9)
Functional mobility—Timed Up and Go test	80 (65.6)
Functional mobility—30 s chair—stand test	82 (67.2)
Functional mobility—4 Stage Balance Test	67 (54.9)
Functional mobility—All 3 tests	79 (64.8)
Assessment—Multifactorial process	75 (61.5)
Education/intervention—e.g., ways to decrease fall risk based on results and recommendations in algorithms and referrals	105 (86.1)
**When did you first implement STEADI?** **(Question only posed to respondents who answered ‘Yes’ to using the STEADI) (*n* = 111)**	
<1 year ago	14 (7.9)
1–2 year ago	18 (16.2)
2–3 years ago	23 (20.7)
3–4 years ago	14 (12.6)
More than 4 years ago	42 (37.8)
**Was it your choice to integrate STEADI into your practice or an expectation?** **(Question only posed to respondents who answered ‘Yes’ or ‘Other’ to using the STEADI) (*n* = 121)**	
My choice	92 (76.0)
Employer mandate	10 (8.3)
Employer recommendation	13 (10.7)
Other	6 (5.0)
**Is the STEADI screen embedded in your practice/documentation workflow?** **(Question only posed to respondents who answered ‘Yes’ or ‘Other’ to using the STEADI) (*n* = 121)?**	
Yes	52 (43.0)
No	63 (52.1)
Unsure	3 (2.5)
Other	3 (2.5)

Note. Percentages are rounded to one decimal place and may total greater than 100% due to rounding. Stopping Elderly Accidents, Deaths, and Injuries (STEADI).

## Data Availability

The data are not publicly available.
